# Impact of the FreeStyle Libre 2^®^ System on Glycaemic Outcomes in Patients with Type 1 Diabetes—Preliminary Study

**DOI:** 10.3390/diagnostics14161777

**Published:** 2024-08-15

**Authors:** Katarzyna Rutkowska, Agnieszka Łoś-Stegienta, Michał Bagiński, Ewa Zięba, Adrianna Baran, Monika Żurawska-Kliś, Marcin Kosiński, Katarzyna Cypryk

**Affiliations:** 1 Department of Internal Diseases and Diabetology, Medical University of Lodz, 92-213 Lodz, Poland; 2 Faculty of Medicine, Medical University of Lodz, 90-419 Lodz, Poland

**Keywords:** type 1 diabetes mellitus, flash glucose monitoring, glycaemic control

## Abstract

We aimed to evaluate glycaemic control in patients with type 1 diabetes during the first three months of use of the flash glucose monitoring (FGM) system. Methods: We conducted a study of a cohort of 81 people with type 1 diabetes mellitus who used the FreeStyle Libre 2 (FSL2) sensor continuously for 3 months. Patients had not used a CGM before. The effectiveness of using the FSL2 system was assessed using AGP reports at two time points (3–4 weeks and 11–12 weeks of system use). Results: Eight weeks after using FSL2, compared with results from 3–4 weeks of use, there were no differences in the glucose management indicator, time spent in range, above range and below range, or glucose variability. In the first month of FGM use, patients scanned the sensor significantly more often than in the following two months (*p* = 0.021). No significant differences were found in the change of the evaluated parameters when comparing patients by duration of diabetes and treatment method. Conclusions: Short-term use of FSL2 promotes a significant reduction in GMI in patients with more time spent in hyperglycaemia (especially > 250 mg/dL). In this short period of use, no other changes in glycaemic control parameters are observed.

## 1. Introduction

Regular glycaemic control is one of the most important aspects of the management of patients with diabetes, especially type 1. It enables appropriate therapeutic intervention aimed at achieving a good metabolic control of diabetes, thus reducing the risk of hypoglycaemic and hyperglycaemic episodes, and preventing the development of serious complications that can lead to premature deterioration of health and reduced quality of life [1,2].

One of the major problems in diabetes management is infrequent blood glucose monitoring. The need for very frequent blood glucose measurements using glucometers (self-monitoring of blood glucose, SMBG) is a fundamental limitation in the ability to properly implement functional insulin therapy. Patients often cite various limitations associated with blood glucose meters, such as pain, time, invasiveness and lack of discretion, as the main reasons for limited self-monitoring [3].

The solution to this problem lies in the use of continuous glucose monitoring (CGM) systems. Modern technologies, which have been available in Poland and worldwide for several years, allow patients to monitor their glucose levels very accurately, significantly improving metabolic control and the quality of life of both patients and caregivers [4,5,6].

Changes in reimbursement policy in Poland, effective from 1 January 2023, regarding flash glucose monitoring (FGM) systems for patients over 18 years of age requiring intensive insulin therapy have enabled diabetes patients to have wider access to modern glucose monitoring methods. It will be interesting to see whether the amount of time with access to CGM will have a positive impact on glycaemic parameters in the short term.

This study aimed to assess whether changing from SMBG to FGM would result in improved parameters describing the degree of glycaemic control. The study also aimed to evaluate which patients would benefit most from the use of CGM systems based on age, duration of diabetes and treatment method.

## 2. Materials and Methods

This study included 81 patients with type 1 diabetes who used the FreeStyle Libre 2^®^ (FSL2) sensor continuously for 3 months. Regarding gender, 51% of the participants were female. The mean age was 41 ± 12 years, the mean duration of diabetes was 21 ± 12 years, and 58% of the participants had had diabetes for more than 10 years. For treatment, 59% of the patients were treated with multiple daily injections (MDI), while the rest were treated with continuous subcutaneous insulin infusion (CSII). The mean BMI was 25.5 ± 3.9 kg/m^2^. All patients were treated at the Outpatient Department of Diabetology, Lodz, Poland. During the initiation of the FSL2 system, patients were not given information on how to interpret and handle the data obtained. They were given access to the product and technical instructions on how the FGM system works.

The effectiveness of the FSL2 system was based on the assessment of parameters included in the Ambulatory Glucose Profile (AGP) reports developed by the International Diabetes Center (IDC), using data from two time periods (period A: 3–4 weeks and period B: 11–12 weeks of system use). Based on the conclusions of the International Consensus, glycaemic values were categorised as target range (TIR, 70–180 mg/dL), above range (TAR, >180 mg/dL and >250 mg/dL) and below range (TBR, <70 mg/dL and <54 mg/dL) [7]. Data from electronic medical records (medical history and anthropometric measurements) were also collected.

Patients were divided into groups according to age (<40 years vs. ≥40 years), duration of diabetes (<10 years vs. ≥10 years) and treatment method (MDI vs. CSII). The change in parameters was calculated as the difference between the value at week 11–12 and the value at week 3–4. Patients were divided at the age of 40 years, based on the assumption that this age spans two central decades of average life expectancy. According to Statistics Poland, the average life expectancy in 2023 was approximately 75 years for men and 82 years for women, making 40 years roughly the midpoint of life expectancy. 

All collected data were statistically analysed. Statistical significance was set at the *p* < 0.05 level. Statistical analyses were performed using Statistica 13.3 software (USA) and Microsoft 365 Excel version 2407 (USA).

## 3. Results

Baseline characteristics are shown in Table 1.

### 3.1. Glycaemic Results

Mean sensor glucose concentration and glucose management indicator (GMI) did not differ between the time points studied (*p* = 0.856 and *p* = 0.882, respectively). There were no differences in TIR, TAR and TBR values, presented as percentages and in minutes, between the time points evaluated. Patients experienced low glucose events with similar frequency at both time points. During the first month of FGM use, patients scanned the sensor significantly more often compared to weeks 11 and 12 of system use. (13.5 ± 7.9 vs. 11.9 ± 7.4, *p* = 0.021). Results are shown in Table 2.

### 3.2. Change in Glycaemic Results by Group

Patients aged <40 years had significantly fewer readings from the sensor in the following weeks of system use (*p* = 0.007). Their glycaemic control was worse compared to patients aged ≥40 years, with mean glucose, GMI and TAR increasing and TIR decreasing. However, the differences did not reach statistical significance. In the group of patients aged <40 years, a non-significant decrease in glycaemic variability was observed. Table 3 shows the results as changes in parameters between the second and first assessment periods.

No significant differences were found in the change of the evaluated parameters during the 8 weeks of FGM use, distinguishing patients according to duration of diabetes and treatment method (Table 4).

### 3.3. Change in GMI

During the 8 weeks of use of the FGM system, 12 patients (15%) reduced their GMI by at least 0.5%. They had a significantly higher mean glucose concentration (166 ± 35 mg/dL vs. 148 ± 26 mg/dL, *p* = 0.043) and a significantly higher percentage of time spent in hyperglycaemia >250 mg/dL (12 ± 10% vs. 7 ± 6%, *p* = 0.029) during weeks 3 and 4 of system use compared to patients whose GMI did not improve by at least 0.5%. There were no statistically significant differences between the groups in any of the other parameters assessed.

## 4. Discussion

Our study showed that the time with access to CGM has no effect on glycaemic parameters in the short term of use. We did not demonstrate significant differences during 8 weeks of FSL2 use in the glycaemic results of patients with T1DM.

A meta-analysis of 19 studies involving 2013 participants showed that the use of CGM systems significantly improved outcomes in achieving treatment goals and reduced the incidence of hypoglycaemic episodes [8]. In 108 patients with type 1 diabetes who were previously using FSL (without low and high glucose alarms), the frequency of hypoglycaemic episodes was significantly reduced after 12 weeks of using the FSL2 system [9]. Similar results were found by Boscari et al. [10]. The cited patients had already used the FGM system, so continuous monitoring was not a new technology for them. In addition, they were instructed not only how to use the device, but also how to interpret and respond to low and high glucose alarms. The education program also focused on the ability to correct hyperglycaemia, which may have been missing in our observation.

The use of FGM in adolescents with type 1 diabetes contributed to an improvement in HbA1c after 6 months of use, and this effect was maintained after 18 months of use [11]. Similarly, in a study by Hayek et al., the use of FSL contributed to improvements in metabolic control and quality of life in adolescents with diabetes [12]. The beneficial effect of the FGM system was evident after 3 months of use. In both studies, patients received detailed instructions on how to use the FGM system and underwent specific training. Ongoing patient education on how to interpret the data obtained from the FGM system is necessary to take advantage of its full potential. A member of the diabetes team (physician, educator or nurse) should educate the patient each time the AGP report is analysed [13]. The authors of the study point out that it is important to discuss with the patient his expectations of the system and explain how it works (including possible differences in glucose values compared to SMBG, and the delay in readings compared to SMBG). It is also important to learn how to interpret the results in a practical way.

In the COMISAIR study, which included 94 participants with type 1 diabetes, improvement in the metabolic control of diabetes was achieved after the use of CGM systems in patients treated with both insulin pumps and pens [14]. The use of CGM was significant in reducing HbA1c compared to the use of glucometers. The method of treatment (MDI vs. CSII) did not affect the reduction in HbA1c levels. Similarly, in our study, no differences in glycaemic outcomes were observed according to the therapy used (MDI vs. CSII).

The FLARE-NL 5 study showed that of the variables analysed (age, sex, baseline HbA1c, quality of life survey results), only a high baseline HbA1c level had a significant effect on the reduction of HbA1c levels when using FSL [15]. The study included 860 participants (75% with type 1 diabetes). Of these, 187 (22%) had a significant reduction in HbA1c (≥0.5). In our study, a similar percentage of patients (15%) reduced their GMI by at least 0.5%. We also found no effect of patient age on the glycaemic parameters with FGM use.

In the large FUTURE study published in Diabetes Care in 2020, one year after the reimbursement of the scanning CGM system, a stabilisation of metabolic control of diabetes, a reduction in the number of hypoglycaemic episodes and a reduction in the cost to the insurer of hospitalisation for acute complications of the disease were observed [16]. A total of 1913 people who had been living with type 1 diabetes for more than 3 months were included in the study. None of the participants had used a CGM before the study. Patients were trained in setting up the device and interpreting trend arrows and glucose sensor reports. HbA1c was slightly lower at 6 months (7.7%) compared to baseline (7.8%; *p* < 0.0001), with a return to the baseline at one year (7.8%; *p* = 0.287). In our study, the HbA1c did not change either, but we assessed a much shorter period of time (8 weeks).

Different results were found in a meta-analysis conducted in 2022 [17]. The analysis included 28,063 adults and children with type 1 diabetes from 62 real-world studies. After 3–4 months of using the FSL system, HbA1c in adults with type 1 diabetes decreased by −0.53% (*p* < 0.0001). The beneficial effects of using the FGM system were maintained at 12 and 24 months after its introduction. We recognise the need for further studies to assess the effectiveness of the FSL2 system in improving glycaemic outcomes in longer perspectives in Polish patients with diabetes.

A major strength of this study is the homogenous group of patients. The study was conducted in a centre with many years of experience in the management of type 1 diabetes. This is the first Polish study known to the authors to evaluate the impact of the time with access to the FGM system on the glycaemic outcomes in patients with diabetes.

The potential limitation of this study is its single-centre design. The study was relatively short and the results need to be confirmed with a longer follow-up period. In addition, the use of FSL2 was assessed in a group of patients with initially satisfactory glycaemic control, which may have influenced the results.

## 5. Conclusions

In the short period of use of the FGM system, no changes in glycaemic control parameters were observed. None of the groups studied showed a significant benefit of the use of the FGM system. Short-term use of FSL2 promotes a significant reduction in GMI in patients with more time spent in hyperglycaemia (especially those > 250 mg/dL).

We speculate that the decreasing number of scans per day over time with the use of CGM is a result of “habituation” or waning interest in the device as a “technical novelty”, which should be addressed.

Further randomised, prospective studies and the development of a structured patient education system are needed to realise the full potential of CGM’s capabilities.

## Figures and Tables

**Table 1:** Baseline characteristics.

Baseline Characteristics	Value n = 81
Male [n/%]	40/49
Female [n/%]	41/51
Age [years]	41 ± 12
Duration of diabetes [years]	21 ± 12
BMI [kg/m^2^]	25.5 ± 3.9
MDI [n/%]	48/59
SCII [n/%]	33/41

BMI—Body Mass Index, MDI—multiple daily injections, CSII—continuous subcutaneous insulin infusion.

**Table 2:** Glycaemic outcomes.

Variables	Period A 3–4 Weeks of FGM	Period B 11–12 Weeks of FGM	*p*
Average glucose [mg/dL]	150 ± 28	151 ± 27	0.856
Daily scans	13.5 ± 7.9	11.9 ± 7.4	0.021
GMI [%]	6.92 ± 0.67	6.92 ± 0.65	0.882
Glucose variability [%]	35.6 ± 7.62	34.8 ± 6.92	0.110
TIR [%]	67.6 ± 16.7	68.4 ± 15.9	0.377
TAR > 180 mg/dL [%]	20.09 ± 11.3	19.7 ± 10.5	0.560
TAR > 250 mg/dL [%]	7.46 ± 7.54	7.50 ± 7.75	0.951
TBR < 70 mg/dL [%]	3.92 ± 3.53	3.72 ± 3.38	0.545
TBR < 54 mg/dL [%]	0.85 ± 1.94	0.54 ± 1.54	0.219
Low glucose events [n]	8.9 ± 6.8	8.6 ± 7.1	0.697

GMI—glucose management indicator, TIR—time in range, TAR—time above range, TBR—time below range.

**Table 3:** Changes in glycaemic parameters between period A and B in groups according to age.

Variables	Age < 40 yrs. n = 44	Age ≥ 40 yrs. n = 37	*p*
Average glucose [mg/dL]	3.25 ± 15.8	−3.18 ± 14.9	0.05
Daily scans	−2.77 ± 4.36	−0.14 ± 4.16	0.007
GMI [%]	0.07 ± 0.38	−0.07 ± 0.36	0.08
Glucose variability [%]	−1.49 ± 4.37	0.09 ± 4.03	0.09
TIR [%]	−0.63 ± 8.62	2.57 ± 7.86	0.08
TAR > 180 mg/dL [%]	0.34 ± 5.69	−1.19 ± 5.25	0.215
TAR > 250 mg/dL [%]	1.0 ± 5.33	−1.11 ± 5.53	0.08
TBR < 70 mg/dL [%]	−0.48 ± 2.72	0.14 ± 3.15	0.351
TBR < 54 mg/dL [%]	−0.23 ± 1.59	−0.41 ± 2.85	0.724
Low glucose events [n]	−0.5 ± 6.15	0 ± 6.45	0.722

GMI—glucose management indicator, TIR—time in range, TAR—time above range, TBR—time below range.

**Table 4:** Changes in glycaemic parameters between period A and B in groups according to DM duration and treatment method.

Variables	DM Duration <10 yrs. n = 34	DM Duration ≥10 yrs. n = 47	*p*	MDI n = 48	CSII n = 33	*p*
Average glucose [mg/dL]	2.48 ± 16.0	−0.35 ± 14.9	0.422	1.9 ± 16.1	−0.32 ± 15.4	0.556
Daily scans	−1.94 ± 4.32	−1.41 ± 4.58	0.607	−1.85 ± 3.87	−2.0 ± 5.2	0.870
GMI [%]	0.05 ± 0.38	−0.004 ± 0.36	0.509	0.04 ± 0.39	−0.01 ± 0.36	0.506
Glucose variability [%]	−1.14 ± 4.17	−0.66 ± 4.39	0.625	−1.10 ± 4.17	−0.60 ± 4.79	0.643
TIR [%]	0.09 ± 8.66	0.82 ± 7.76	0.693	0.76 ± 8.44	0.28 ± 8.72	0.816
TAR > 180 mg/dL [%]	−0.06 ± 5.80	−0.46 ± 5.45	0.757	−0.47 ± 5.89	0.10 ± 5.42	0.677
TAR > 250 mg/dL [%]	1.03 ± 5.13	−0.22 ± 5.02	0.284	0.62 ± 4.94	0.32 ± 5.65	0.464
TBR < 70 mg/dL [%]	−0.757 ± 2.46	0.152 ± 3.23	0.178	−0.54 ± 3.36	0.17 ± 2.6	0.339
TBR < 54 mg/dL [%]	−0.30 ± 1.31	−0.30 ± 2.78	0.998	−0.357 ± 2.8	−0.25 ± 1.69	0.857
Low glucose events [n]	−1.12 ± 4.18	0.28 ± 7.50	0.334	−1.11 ± 6.47	0.75 ± 6.53	0.242

GMI—glucose management indicator, TIR—time in range, TAR—time above range, TBR—time below range.

## Data Availability

The datasets presented in this article are not readily available because the data are part of an ongoing study. Requests to access the datasets should be directed to the corresponding author (K.R.).
